# Efficacy and safety of Q-switched laser combined with intense pulsed light in treating melasma: a meta-analysis

**DOI:** 10.3389/fmed.2026.1749950

**Published:** 2026-02-12

**Authors:** Yajun Zhang, Jianxin Zheng, Huijuan Wang, Xiaoye Liu, Lijuan Liu, Guoqiang Zhang

**Affiliations:** 1 Department of Dermatology, The First Hospital of Hebei Medical University, Shijiazhuang, Hebei, China; 2 Hebei Technical Innovation Center for Dermatology and Medical Cosmetology Technology, Shijiazhuang, Hebei, China; 3 Shijiazhuang Boyaman Medical Aesthetic Clinic, Shijiazhuang, Hebei, China

**Keywords:** efficacy, intense pulsed light, melasma, meta-analysis, Q-switched laser, safety

## Abstract

**Background:**

Melasma is a common dermatological disorder characterized by hyperpigmented facial patches, which significantly impacting patients' quality of life and imposing substantial economic burdens. Given the multifaceted impact of melasma, there is a pressing need for effective therapeutic strategies. This meta-analysis evaluates the efficacy and safety of combining Q-switched (QS) laser with intense pulsed light (IPL) vs. monotherapy for melasma.

**Methods:**

A systematic literature search of relevant databases was conducted to identify controlled trials comparing the combination therapy with monotherapy. Primary outcomes included changes in the Melasma Area and Severity Index (MASI) score and adverse reactions. Secondary outcomes comprised clinical efficacy rate, recurrence rate, and patient satisfaction rate. Data were pooled using a fixed-effects model.

**Results:**

A total of 31 studies involving 2,801 patients were included. Results showed that the combination therapy demonstrated a significantly greater reduction in MASI scores than monotherapy (standardized mean difference: 1.20, 95% CI: −1.36 to −1.05; *p* < 0.001). The pooled efficacy rate was also higher for the combination group (92.0% vs. 77.4%; risk ratio: 1.10, 95% CI: 1.03–1.17; *p* < 0.05). Moreover, the combination group exhibited a lower recurrence rate (8.5 vs. 17.8%; risk ratio: 0.54, 95% CI: 0.32–0.90; *p* < 0.05). However, no significant difference in patient satisfaction was observed between the two groups. The incidence of adverse events was comparable between the treatments.

**Conclusion:**

This meta-analysis indicates that Q-switched laser combined with intense pulsed light results in greater objective improvement in melasma than monotherapy, without an increased risk of adverse events. In contrast, patient satisfaction shows no significant improvement, suggesting that patient-reported outcomes should be considered alongside clinician-assessed measures. Additional longitudinal studies are needed to establish sustained efficacy and clarify patient-perceived benefits to further refine clinical protocols.

**Systematic review registration:**

https://www.crd.york.ac.uk/prospero/display_record.php?ID=CRD420251170319, identifier: CRD420251170319.

## 1 Introduction

Melasma is a common, acquired hyperpigmentation disorder that presents a significant clinical challenge due to its chronic nature, high relapse rates, and pronounced impact on patients' quality of life (1, 2). This condition disproportionately affects women and individuals with darker skin phototypes (Fitzpatrick III–VI) (3, 4). The pathophysiology of melasma is multifactorial and complex, involving not only the hyperactivity of melanocytes but also the influence of ultraviolet radiation, hormonal factors, underlying vascular and inflammatory components, and disruption of the basement membrane (3, 4). These intertwined mechanisms contribute to its resistance to treatment and high recurrence rates. Current first-line therapies, including topical agents (e.g., triple-combination creams) and systemic treatments (e.g., tranexamic acid), are often limited by their efficacy, adverse effects, and propensity for high recurrence rates (3, 5).

Laser and light-based modalities, such as Q-switched (QS) lasers and intense pulsed light (IPL), have emerged as valuable options for refractory cases. Their complementary mechanisms—with QS lasers targeting melanin via selective photothermolysis and IPL targeting both pigmentary and vascular components—provide a rationale for combination therapy aimed at simultaneously addressing multiple pathogenic pathways (4, 6, 7). This potential synergy suggests that combining QS lasers, which target melanin via selective photothermolysis, with IPL, which targets both pigmentary and vascular components, may improve outcomes and reduce relapse rates by simultaneously targeting epidermal and dermal pigments (4, 6–8). However, monotherapy with these modalities can be problematic due to inconsistent response rates, a significant risk of post-inflammatory hyperpigmentation (PIH) especially in patients with darker skin types (Fitzpatrick III–VI), and the requirement for multiple treatment sessions (9, 10).

Critically, there is a distinct gap in the current evidence base. While narrative reviews discuss combination therapies, there is a lack of comprehensive quantitative synthesis specifically comparing the combined efficacy and safety of QS laser and IPL against each modality alone. Existing meta-analyses have typically focused on single modalities, leaving the relative benefit of this specific combination unclear. Furthermore, available comparative studies are limited by methodological heterogeneity, small sample sizes, and often insufficient follow-up to assess relapse rates. Therefore, a rigorous synthesis of efficacy and safety data is urgently needed to guide clinical decision-making.

It is also crucial to consider the study population's context. The risk of PIH and treatment response varies significantly with skin phototype (9). Notably, the majority of the existing primary research on laser and light-based therapies for melasma, and consequently the evidence base for this meta-analysis, has been conducted in populations with Fitzpatrick skin types III–V, predominantly comprising individuals of East Asian descent (4, 9, 10). This demographic focus must be acknowledged upfront as it directly impacts the safety profile and generalizability of the findings to other ethnic groups and skin phototypes.

Therefore, this study employs a systematic review and meta-analysis to quantitatively evaluate the efficacy—measured by reduction in the Melasma Area and Severity Index (MASI) score, a tool assessing the extent and severity of melasma—and safety of combining QS laser with IPL vs. either modality alone. To address the identified gaps, this research provides the first focused meta-analysis comparing this specific combination against monotherapies, synthesizing data from both randomized controlled trials and non-randomized controlled trials to offer a comprehensive, quantitative evaluation. Through this comprehensive evidence synthesis, our study aims to optimize therapeutic strategies and improve care for patients suffering from this challenging condition.

## 2 Materials and methods

### 2.1 Data source and search

We conducted a comprehensive literature search in accordance with the PRISMA guidelines. The search spanned multiple electronic databases from their inception until September 13, 2025. The databases searched included both English and Chinese language sources: PubMed, Embase, and the Cochrane Library for English; China National Knowledge Infrastructure (CNKI), VIP Chinese Journal Service Platform, and Wanfang Data Knowledge Service Platform for Chinese. Our objective was to identify all relevant studies on the application of Q-switched lasers and intense pulsed light (IPL) for treating melasma. To illustrate, a representative search strategy used in Chinese databases was: (“Q-switched laser” OR “intense pulsed light”) AND “melasma.” Key search terms, such as “melasma,” “chloasma,” “Q-switched laser,” and “intense pulsed light,” were applied to titles, abstracts, and keywords across all databases. The search strategy also included potential variations in terminology, such as “pulsed lasers,” to ensure comprehensiveness. Two researchers independently searched, screened studies, and extracted data; any discrepancies were resolved through discussion with a third researcher to reach consensus.

### 2.2 Inclusion/exclusion criteria

Inclusion criteria: (i) adults (18 +) with melasma, who had undergone no treatments in previous 3 months; (ii) experimental group received combined Q-switched laser and IPL; (iii) control group received either Q-switched laser or IPL; (iv) at least one outcomes included MASI score, efficacy, recurrence, adverse events, or satisfaction; and (v) RCTs or clinical trials with a follow-up duration of at least 3 months.

Exclusion criteria: (i) non-adults; (ii) recent skin treatments; (iii) case series/reports; (iv) no specified outcomes; (v) studies with insufficient follow-up duration of less than 3 months; (vi) literature not published in peer-reviewed journals or lacking full-text access.

### 2.3 Data extraction and quality assessment

We assessed bias risk in 26 randomized controlled trials using the Cochrane Tool (RevMan 5.4.1), and in the five non-randomized controlled trials with the MINORS, categorizing them as low, unclear, or high risk, with disagreements resolved through discussion. The baseline characteristics of the included studies are presented in Table 1. The results of quality assessment are presented in Table 2.

**Table 1:** Characteristics of 31 studies included in meta-analysis.

**References**	**Study type**	**Age (years)**	**Treatment**	**Sample size**	**Frequency**	**Treatment duration**	**Follow up (months)**	**Outcome measures**
Yun et al. (11)	RCT	42.6 ± 1.9	QS + IPL	12	Q2Wk	6 times	4.5	MASI
		43.4 ± 2.0	IPL	12	Q2Wk	6 times	4.5	
Vachiramon et al. (12)	RCT	20–60	QS + IPL	20	QS QWk + IPL Q2We	QS 5 times + IPL 3 times	4	MASI
			QS	20	QS QWe	5 times	4	
Guan et al. (13)	RCT	38.64 ± 7.25	QS + IPL	45	Q4We	4 times	7	MASI, CER, RRate, ARR
		39.27 ± 7.83	IPL	45	Q4We	4 times	7	
Yan et al. (14)	RCT	38.65 ± 2.57	QS + IPL	43	Q4We	6 times	6	MASI
		39.56 ± 2.85	QS	43	Q4We	6 times	6	
Song et al. (15)	NRCT	42.92 ± 12.01	QS + IPL	20	Q3–4We	QS 2 times + IPL 3 times	7	CER, PSR
		43.47 ± 13.13	IPL	20	Q3We	5 times	7	
Gao (16)	RCT	41.02 ± 4.17	QS + IPL	43	Q4We	8 times	10	CER, MASI, PSR, ARR
		41.15 ± 4.20	IPL	42	Q4We	8 times	10	
Shi et al. (17)	NRCT	43.13 ± 5.32	QS + IPL	41	Q4We	10 times	10	CER
		42.84 ± 5.26	IPL	38	Q4We	10 times	10	
Liu et al. (18)	RCT	38.6	QS + IPL	33	Q4We	10 times	10	CER
			IPL	33	Q4We	10 times	10	
Feng et al. (19)	RCT	65.08± 4.17	Q + IPL	45	Q4We	10 times	10	CER, PSR, ARR
		64.15 ± 3.17	IPL	44	Q4We	10 times	10	
Ma (20)	RCT	38.65 ± 6.13	QS + IPL	43	Q4We	6 times	6	CER
		38.72 ± 6.01	IPL	43	Q4We	6 times	6	
Tian and Shao (21)	RCT	36.98± 1.4	QS + IPL	50	Q4We	6 times	6	CER, PSR
		36.43 ± 10.42	QS	50	Q4We	6 times	6	
Liu et al. (22)	RCT	35.5 ± 2.6	QS + IPL	45	Q3We	5–8 times	3–12	CER, MASI, ARR
			QS	45	Q2We	5–10 times	3–12	
Lu (23)	RCT	40.69± 5.63	QS + IPL	8	Q3We	5–8 times	4–6	CER
		40.64± 5.85	QS	7	Q2We	5–10 times	2.5–5	
Lin and Cui (24)	RCT	35.74 ± 5.68	QS + IPL	79	Q2We	10 times	3–12	CER, MASI, ARR, RRate
		34.56 ± 4.27	IPL	79	Q2We	10 times	3–12	
Zhang et al. (25)	RCT	37.04 ± 6.45	QS + IPL	59	QS Q2We + IPL Q8We	QS 6 times + IPL 3 times	12	CER, MASI, RRate
		37.50 ± 6.23	QS	59	Q4We	6 times	12	
Bei (26)	RCT		QS + IPL	60	QS Q4–12We + IPL Q2We	QS 4–6 times + IPL 2–6 times	12	CER, RRate
			QS	60	QS Q4–12We	4–6 times	12	
Yuan (27)	RCT	43.5 ± 4.3	QS + IPL	45	Q4We	5 times	5	CER, PSR
		QS	45	Q4We	5 times	5		
Huang et al. (28)	RCT	27 ± 3.12	QS + IPL	40	QS QWe + IPL Q3–4We	5 times	5	CER
		QS	40	QWe	5 times	5		
Lv (29)	RCT	33.52 ± 2.51	QS + IPL	34	QS Q2–3We + IPL Q3–4We	QS 5 times + IPL 5 times	5	CER, ARR
		33.21± 2.82	QS	34	Q2–3We	5 times	5	
Xiao et al. (30)	RCT	33.7	QS + IPL	42	Q4We	QS 6 times + IPL 5 times	6	CER, ARR
			QS	35	Q4We	6 times	6	
Wu et al. (31)	NRCT	34.28 ± 4.73	QS + IPL	57	Q4We	6 times	6	CER, ARR
		34.28 ± 4.73	IPL	57	Q4We	6 times	6	
Fang et al. (32)	RCT	33.7	QS + IPL	40	Q8We	QS 3 times + IPL 3 times	6	CER, ARR
			QS	32	Q8We	3 times	6	
Qin and Zhang (33)	RCT	41.46 ± 5.74	QS + IPL	40	IPL Q4We + QS QWe	QS 4 times + IPL 3 times	6	CER
		41.23 ± 5.48	IPL	40	Q4We	3 times	6	
Qi (34)	NRCT	38.02 ± 3.46	QS + IPL	63	Q4We	3 times	6	ARR
		35.82 ± 4.09	QS	63	Q4We	3 times	6	
Song (35)	NRCT	42.8 ± 1.5	QS + IPL	36	Q2We	5–10 times	2.5–5	CER
		42.7 ± 1.4	QS	36	Q2We	5–10 times	2.5–5	
Zhang (36)	RCT	20–64	QS + IPL	40	Q8We	QS 3 times + IPL 3 times	6	CER, ARR
		QS	32	Q8We	3 times	6		
Fan (37)	RCT	40.45 ± 2.59	QS + IPL	31	QS Q4We + IPL Q8We	QS 6 times + IPL 3 times	6	CER, ARR
		40.27 ± 2.74	QS	31	Q4We	6 times	6	
Huang (38)	RCT	40.15 ± 6.82	QS + IPL	142	QS Q4We + IPL Q8We	QS 6 times + IPL 3 times	6	CER, ARR
		39.72 ± 6.94	QS	135	Q4We	5 times	5	
Huang (39)	RCT	41.01 ± 4.35	QS + IPL	25	Q4We	6 times	6	MASI, ARR
		39.71 ± 3.08	IPL	25	Q4We	6 times	6	
Liang et al. (40)	RCT	45.84 ± 3.95	QS + IPL	110	Q8We	3 times	6	CER, ARR
		44.84 ± 3.95	QS	110	Q8We	3 times	6	
Liu (41)	RCT	29.7	QS + IPL	30	Q4We	6 times	9	CER, PSR
			QS	25	Q4We	6 times	9	

RCT, randomized controlled trial; NRCT, non-randomized controlled trial; QS, Q-switched laser; IPL, intense pulsed light; MASI, Melasma Area and Severity Indetimes; CER, clinical efficacy rate; RRate, recurrence rate; ARR, adverse reactions rate; PSR, patient satisfaction rate; Q2Wk, once every 2 weeks; Q3Wk, once every 3 weeks; etc.

**Table 2:** The quality assessment of included studies based on methodological index for non-randomized studies (MINORS, range 0–24).

**References**	**Type of study**	**Items**												**Score**	**Quality**
		**1**	**2**	**3**	**4**	**5**	**6**	**7**	**8**	**9**	**10**	**11**	**12**		
Wu et al. (31)	NRCT	2	2	2	2	0	2	2	0	2	2	2	2	20	⋆⋆⋆
Qi (34)	NRCT	2	2	2	2	0	2	2	0	2	2	2	2	20	⋆⋆⋆
Shi et al. (17)	NRCT	2	2	2	2	2	0	2	0	2	2	2	2	20	⋆⋆⋆
Song (35)	NRCT	2	1	2	2	0	0	2	0	2	2	2	2	19	⋆⋆⋆
Song et al. (15)	NRCT	2	2	2	2	0	0	2	0	2	2	2	2	18	⋆⋆⋆

Items are defined as follows (each scored as: 0 = not reported; 1 = reported but inadequate; 2 = reported and adequate): 1. A clearly stated aim. 2. Inclusion of consecutive patients. 3. Prospective collection of data. 4. Endpoints appropriate to the aim of the study. 5. Unbiased assessment of the study endpoint. 6. Follow-up period appropriate to the aim of the study. 7. Loss to follow up less than 5%. 8. Prospective calculation of the study size. 9. An adequate control group. 10. Contemporary groups (control group should be managed during the same period). 11. Baseline equivalence of groups. 12. Adequate statistical analyses. NRCT, non-randomized controlled trial.

### 2.4 Data analysis

Meta-analysis was conducted using Stata 16.0. Continuous variables were reported as standardized mean difference (SMD) and dichotomous variables as relative risk (RR), both with 95% confidence intervals (CI). SMD was preferred over weighted mean difference (WMD) to address two key sources of heterogeneities: variable post-treatment MASI assessment time points (16, 24, 40 weeks) and disparate baseline MASI score distributions (12–21.1 points), ensuring standardized, comparable outcomes. Heterogeneity was assessed using *I*^2^ statistics (fixed-effect model for *I*^2^ ≤ 50% and *p* ≥ 0.1; random-effects model for *I*^2^ > 50% or *p* < 0.1). Publication bias was evaluated through funnel plots, Begg's test, and Egger's test, with *p*-value < 0.05 considered statistically significant.

## 3 Results

### 3.1 Literature screening process and characteristics of included studies

**Figure 1:**
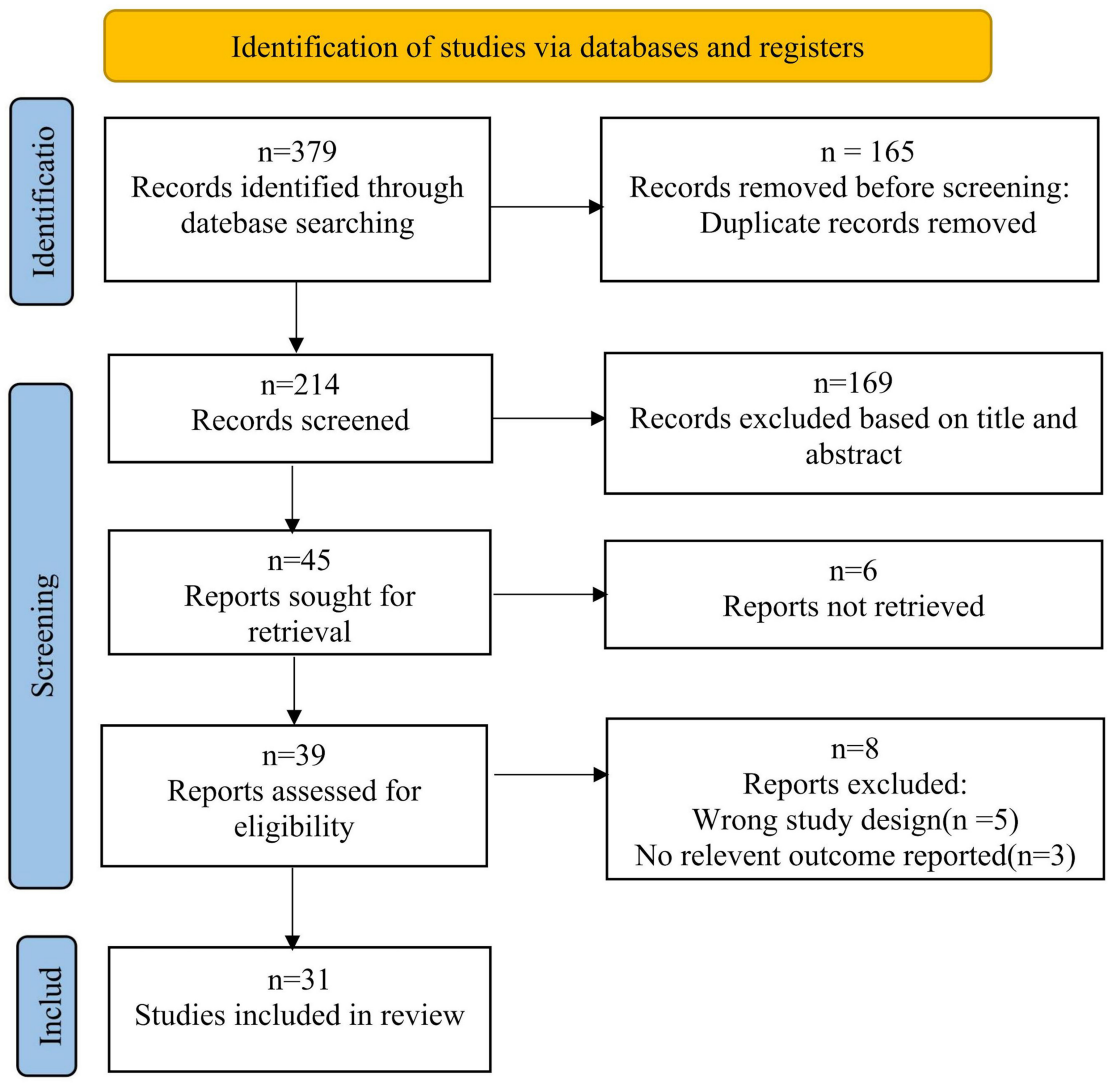
Flow diagram of the study selection. This figure details the process of identifying, screening, assessing eligibility, and including studies in the systematic review.

**Figure 2:**
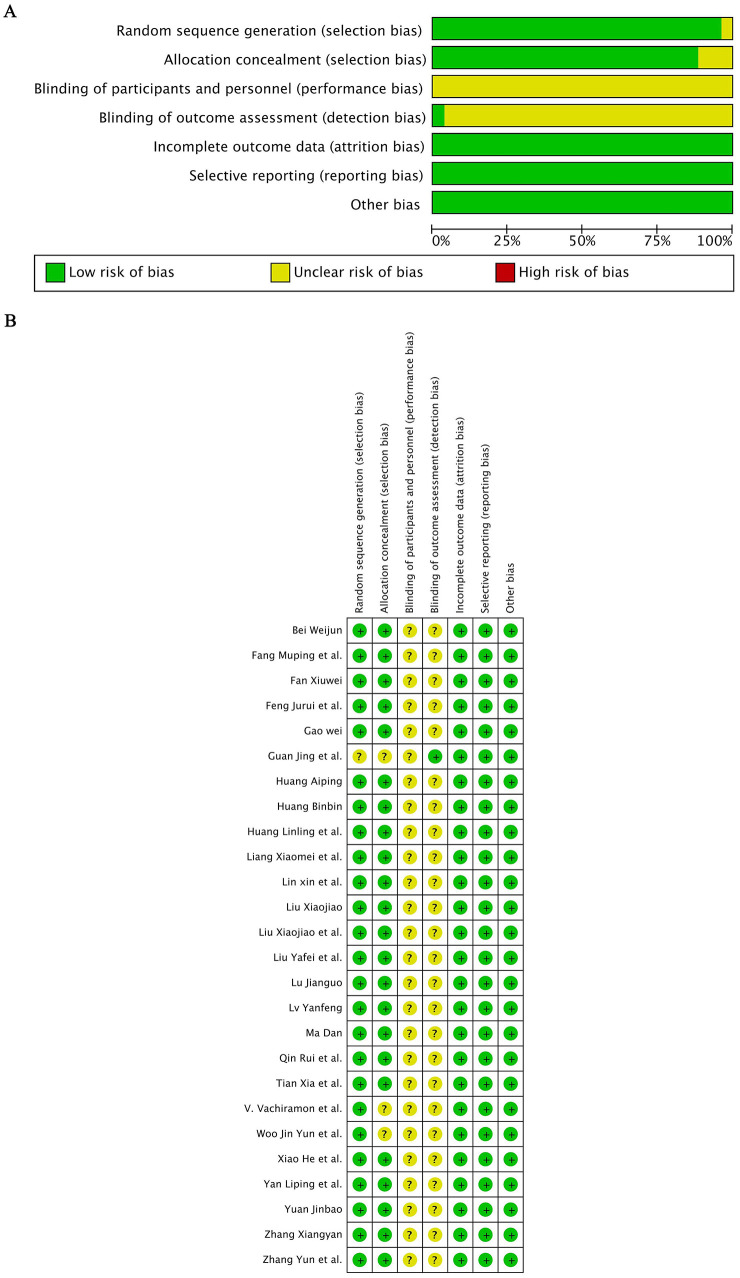
Risk of bias assessment for the included randomized controlled trials. **(A)** Risk of bias summary for each item across all studies. **(B)** Risk of bias graph showing the proportion of studies with low, unclear, or high risk of bias for each domain.

A total of 379 articles were initially identified. After removing 165 duplicates using EndNote X9, 169 records were excluded based on titles and abstracts screening. Six articles were not retrieved, and eight articles were excluded as non-controlled studies or due to incomplete outcome data. Ultimately, 31 studies involving 2,801 patients were included (11–41). The literature screening process is illustrated in Figure 1, and the results of bias risk assessment are illustrated in Figure 2.

### 3.2 Meta-analysis results

Given the uniformly low heterogeneity (*I*^2^ ≈ 0%) observed across multiple pooled outcomes, the fixed-effect model was employed for all meta-analyses as it is statistically justified under this condition. This consistency in heterogeneity may be attributed to the high degree of methodological similarity among the included studies, including comparable treatment protocols (e.g., similar intervention components and dosages), standardized definitions for outcome measures (e.g., clinical efficacy, recurrence), and homogeneous patient selection criteria.

#### 3.2.1 MASI score

**Figure 3:**
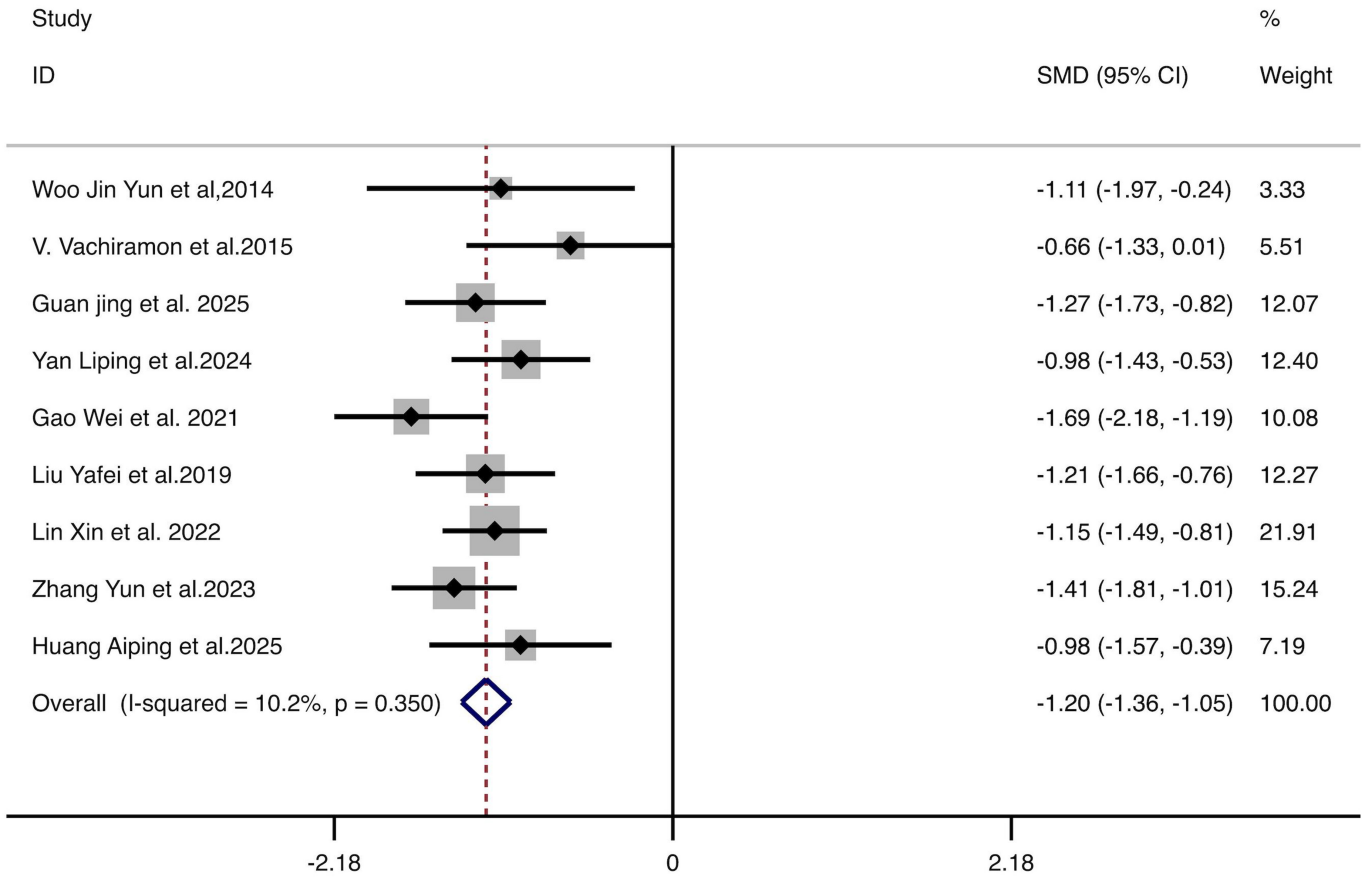
Forest plot for the comparison of MASI score improvement. Each horizontal line represents the SMD and 95% CI for an individual study. The diamond represents the pooled SMD and 95% CI. The vertical line indicates the line of no effect (SMD = 0). The position of the pooled diamond to the left of the vertical line indicates a greater reduction in MASI score for the combination therapy group. MASI, Melasma Area and Severity Index; SMD, standardized mean difference; CI, confidence intervals.

Nine studies reporting the MASI score demonstrated significantly greater improvement in the treatment group compared to controls (SMD = −1.20, 95% CI: −1.36 to −1.05, *p* < 0.001). No significant heterogeneity was observed (*I*^2^ = 10.2%, *p* = 0.350), supporting the use of a fixed-effect model. These results indicate that combination therapy significantly improves melasma severity. The forest plot is shown in Figure 3.

#### 3.2.2 Clinical efficacy rate

In this study, “clinical efficacy rate” referred to the proportion of patients achieving clinical cure, marked effectiveness, or effectiveness combined. The specific criteria included either a post-treatment improvement in MASI score of more than 30%, or a reduction in lesion area exceeding 30% along with significant lightening of pigmentation. The clinical efficacy rate, widely adopted in East Asian clinical research, may have variable definitions and assessment standards across different regions.

**Figure 4:**
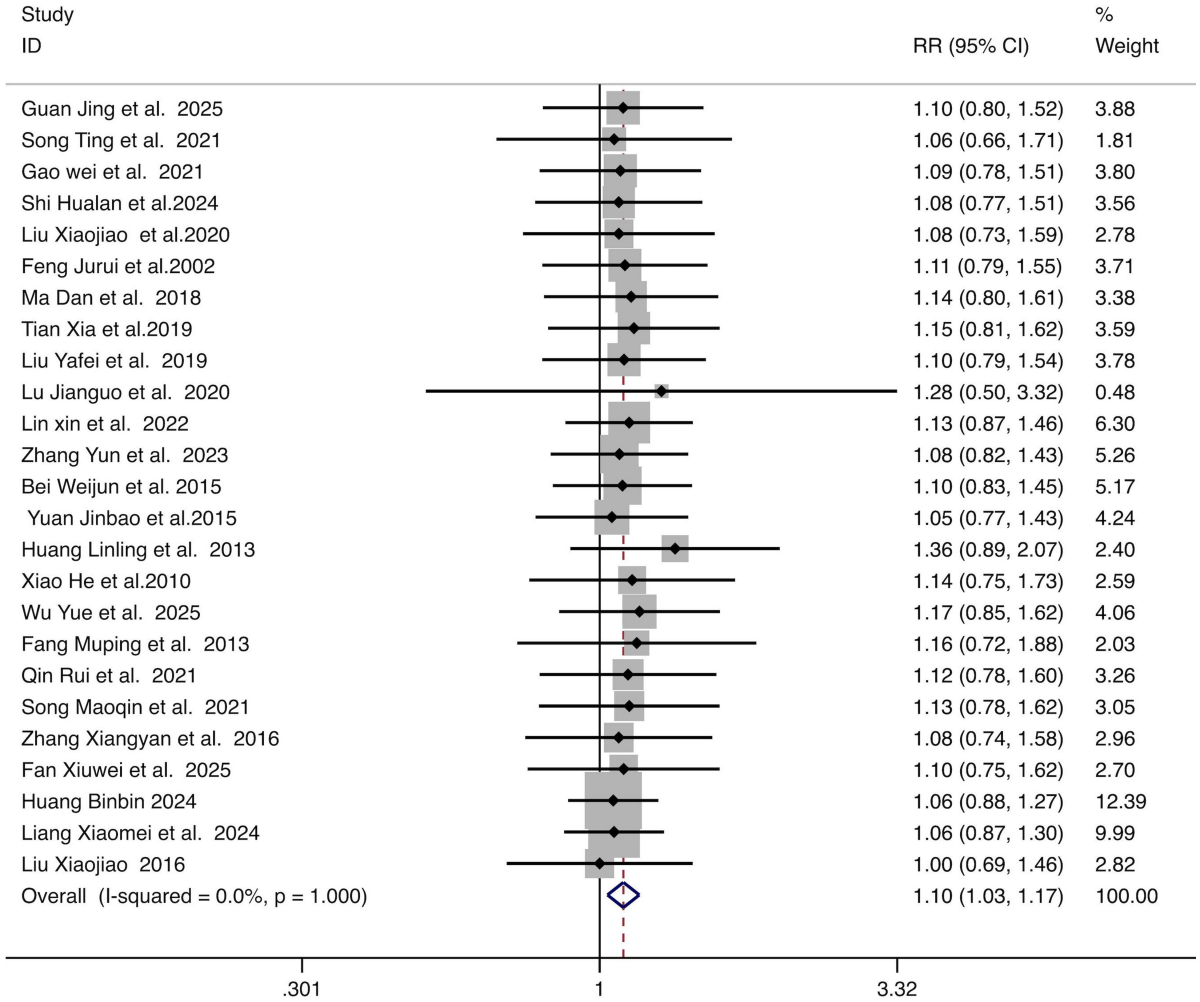
Forest plot for the comparison of clinical efficacy rate. Each horizontal line represents the RR and 95% CI for an individual study. The diamond represents the pooled RR and 95% CI. The vertical line indicates the line of no effect (RR = 1). The position of the pooled diamond to the right of the vertical line indicates a higher efficacy rate in the combination therapy group. RR, relative risk; CI, confidence intervals.

Twenty-six studies reported clinical efficacy rates. With low heterogeneity (*I*^2^ = 0%, *p* = 1.00), a fixed-effect model was used. Combined therapy showed a significantly higher clinical efficacy rate than monotherapy (RR = 1.10, 95% CI: 1.03–1.17, *p* < 0.05). The forest plot is presented in Figure 4.

#### 3.2.3 Recurrence rate

**Figure 5:**
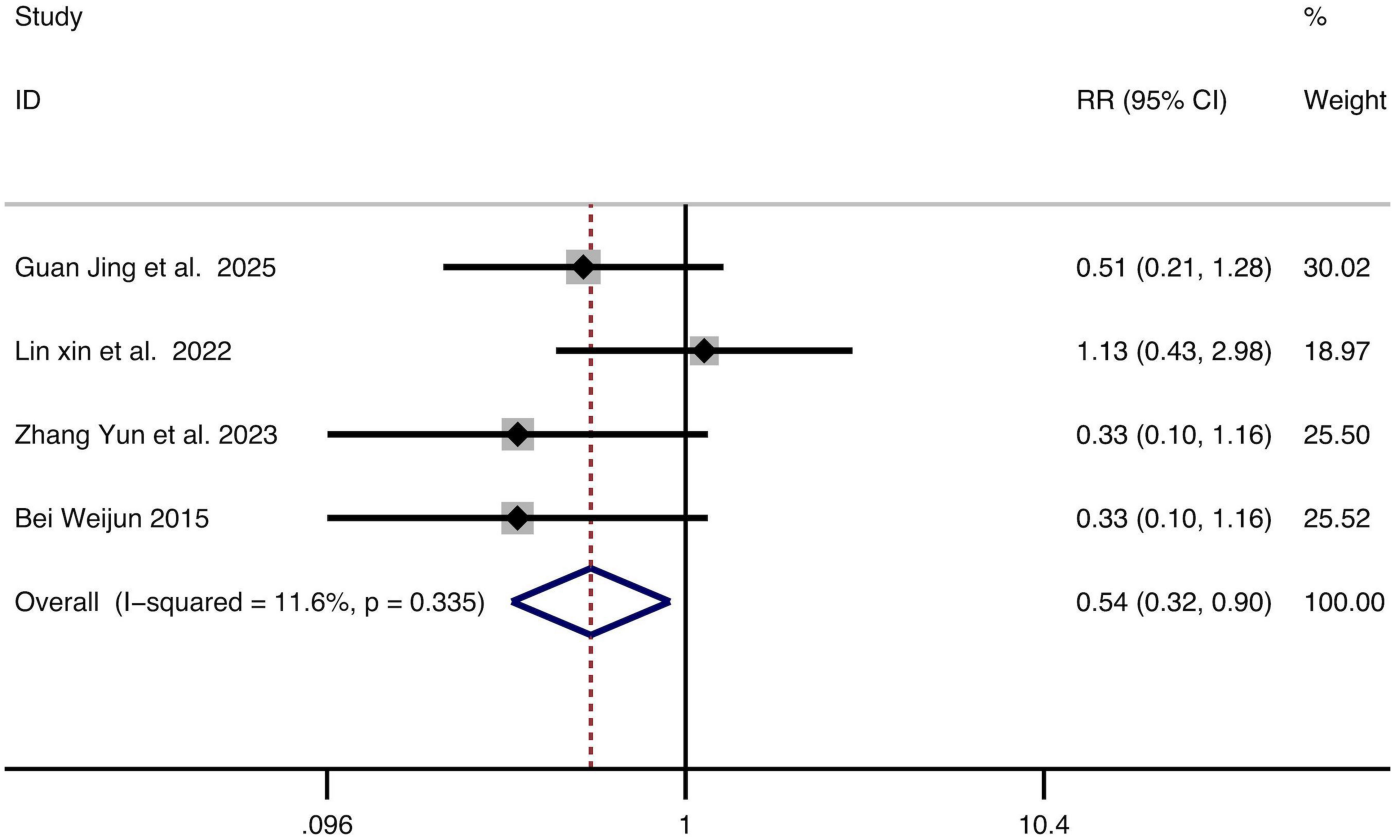
Forest plot for the comparison of recurrence rate. Each horizontal line represents the RR and 95% CI for an individual study. The diamond represents the pooled RR and 95% CI. The vertical line indicates the line of no effect (RR = 1). The position of the pooled diamond to the left of the vertical line indicates a lower recurrence rate in the combination therapy group. RR, relative risk; CI, confidence intervals.

Four studies reported recurrence rates. Given the low heterogeneity (*I*^2^ = 11.6%, *p* = 0.335), a fixed-effect model was used. Combination therapy demonstrated a significantly lower recurrence rate than monotherapy (RR = 0.54, 95% CI: 0.32–0.90, *p* = 0.039). The forest plot is shown in Figure 5.

#### 3.2.4 Adverse reactions rate

**Figure 6:**
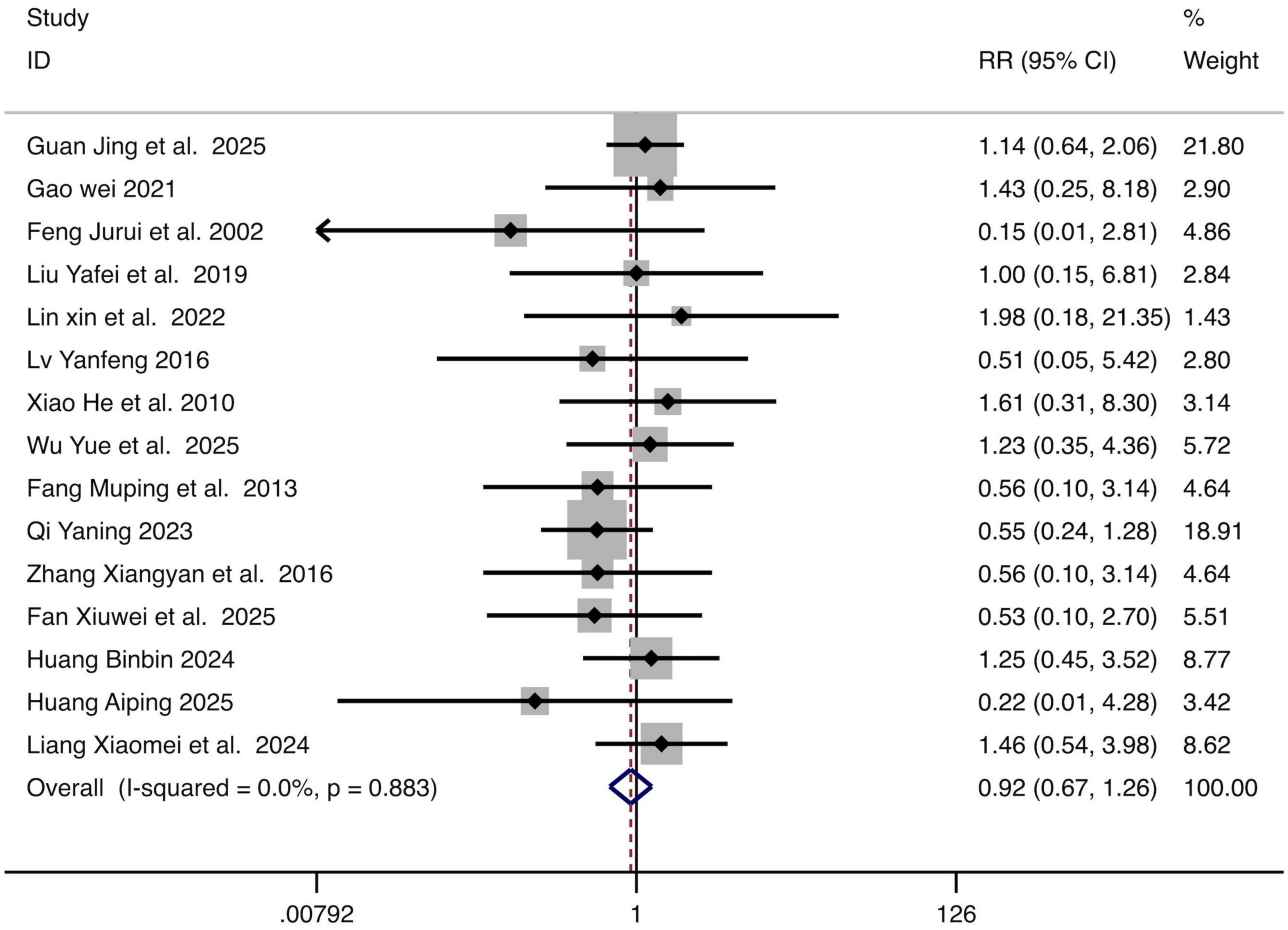
Forest plot for the comparison of adverse reactions rate. Each horizontal line represents the RR and 95% CI for an individual study. The diamond represents the pooled RR and 95% CI. The vertical line indicates the line of no effect (RR = 1). The pooled diamond straddling the vertical line indicates no statistically significant difference between groups. RR, relative risk; CI, confidence intervals.

Fifteen studies reported adverse incidence. With no significant heterogeneity (*I*^2^ = 0%, *p* = 0.883), a fixed-effect model was applied. No significant difference was found in adverse reaction rate between groups (RR = 0.92, 95% CI: 0.67–1.26, *p* = 0.598). The forest plot is presented in Figure 6.

#### 3.2.5 Patient satisfaction rate

**Figure 7:**
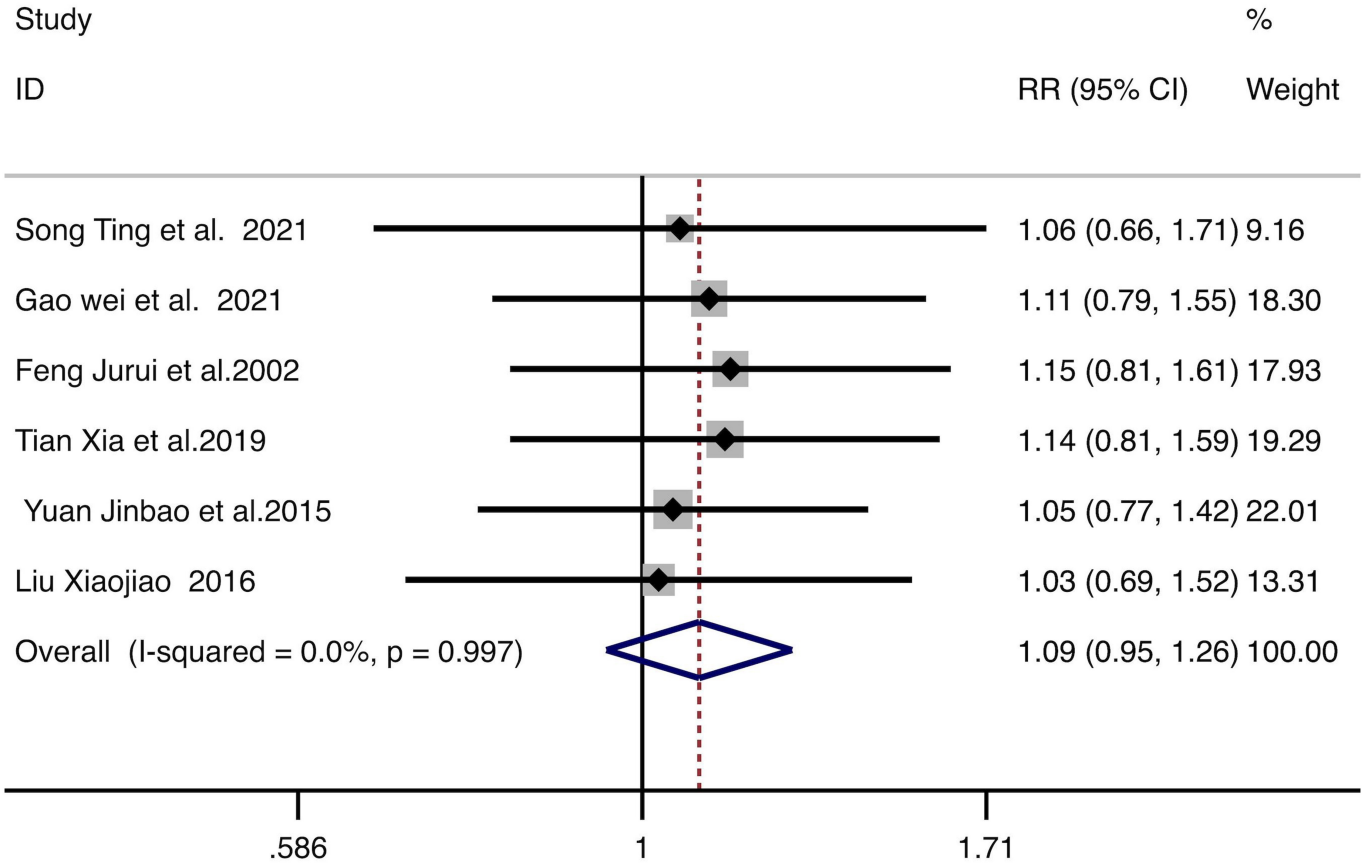
Forest plot for the comparison of patient satisfaction rate. Each horizontal line represents the RR and 95% CI for an individual study. The diamond represents the pooled RR and 95% CI. The vertical line indicates the line of no effect (RR = 1). The pooled diamond straddling the vertical line indicates no statistically significant difference between groups. RR, relative risk; CI, confidence intervals.

Six studies evaluated patient satisfaction rate. With no significant heterogeneity (*I*^2^ = 0%, *p* = 0.997), a fixed-effect model was used. No significant difference was observed between combination therapy and monotherapy (RR = 1.09, 95% CI: 0.95–1.26, *p* = 0.229). The forest plot is shown in Figure 7.

### 3.3 Sensitivity analysis

**Figure 8:**
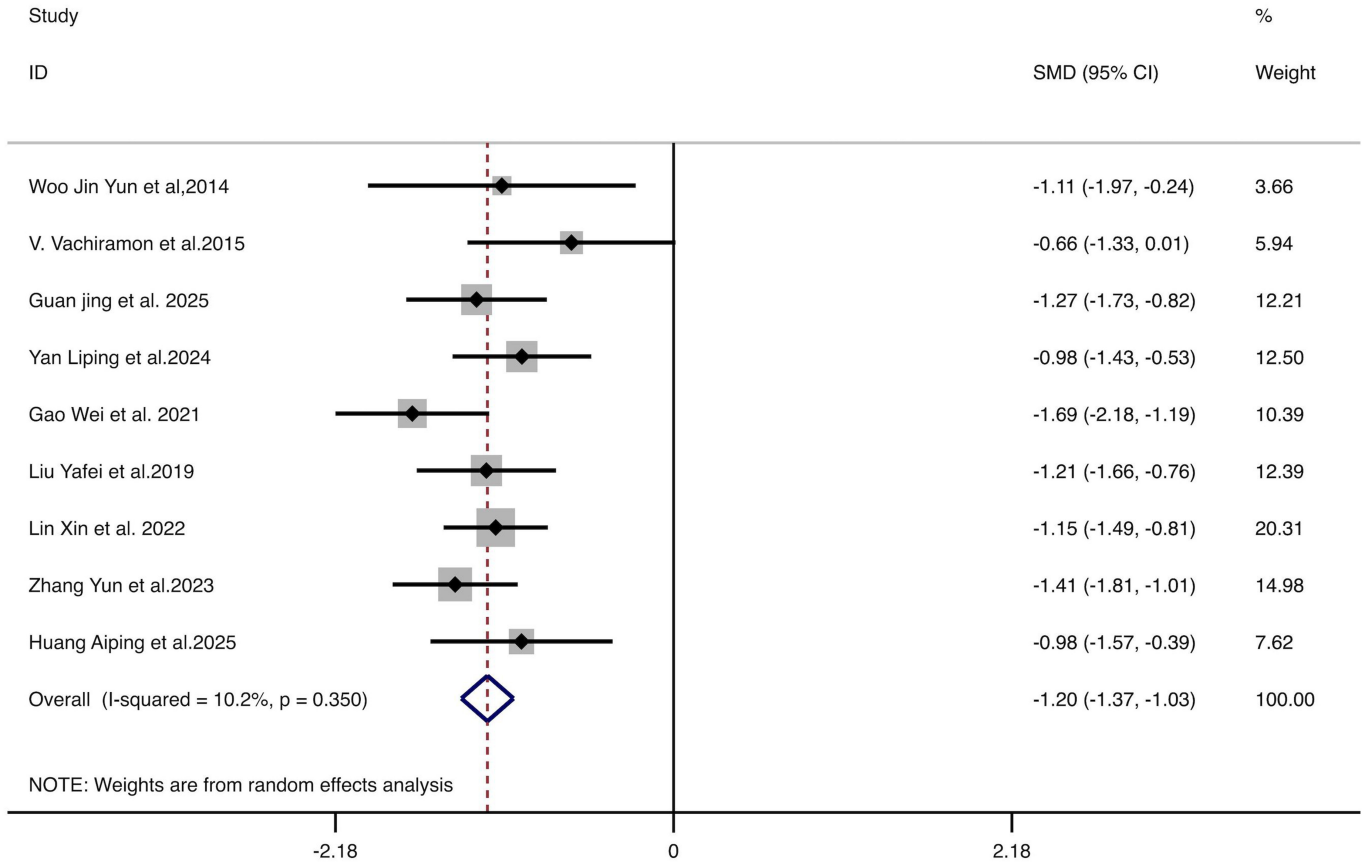
Forest plot for MASI score under the random-effects model (sensitivity analysis). This plot presents the pooled result using the random-effects model, showing consistency with the primary fixed-effect model analysis presented in Figure 3. MASI, Melasma Area and Severity Index; SMD, standardized mean difference; CI, confidence intervals.

A leave-one-out sensitivity analysis was performed initially, where each study was sequentially excluded and the pooled effect size recalculated. No individual study was found to exert a dominant influence on the overall results. Further comparison of fixed-effect and random-effects models under low heterogeneity (*I*^2^ = 10.2%, *p* = 0.350) yielded identical MASI score results (SMD = −1.20, 95% CI: −1.37 to −1.03), confirming the robustness of combination therapy effects regardless of model choice. The forest plot is shown in Figure 8.

## 4 Publication bias assessment

**Figure 9:**
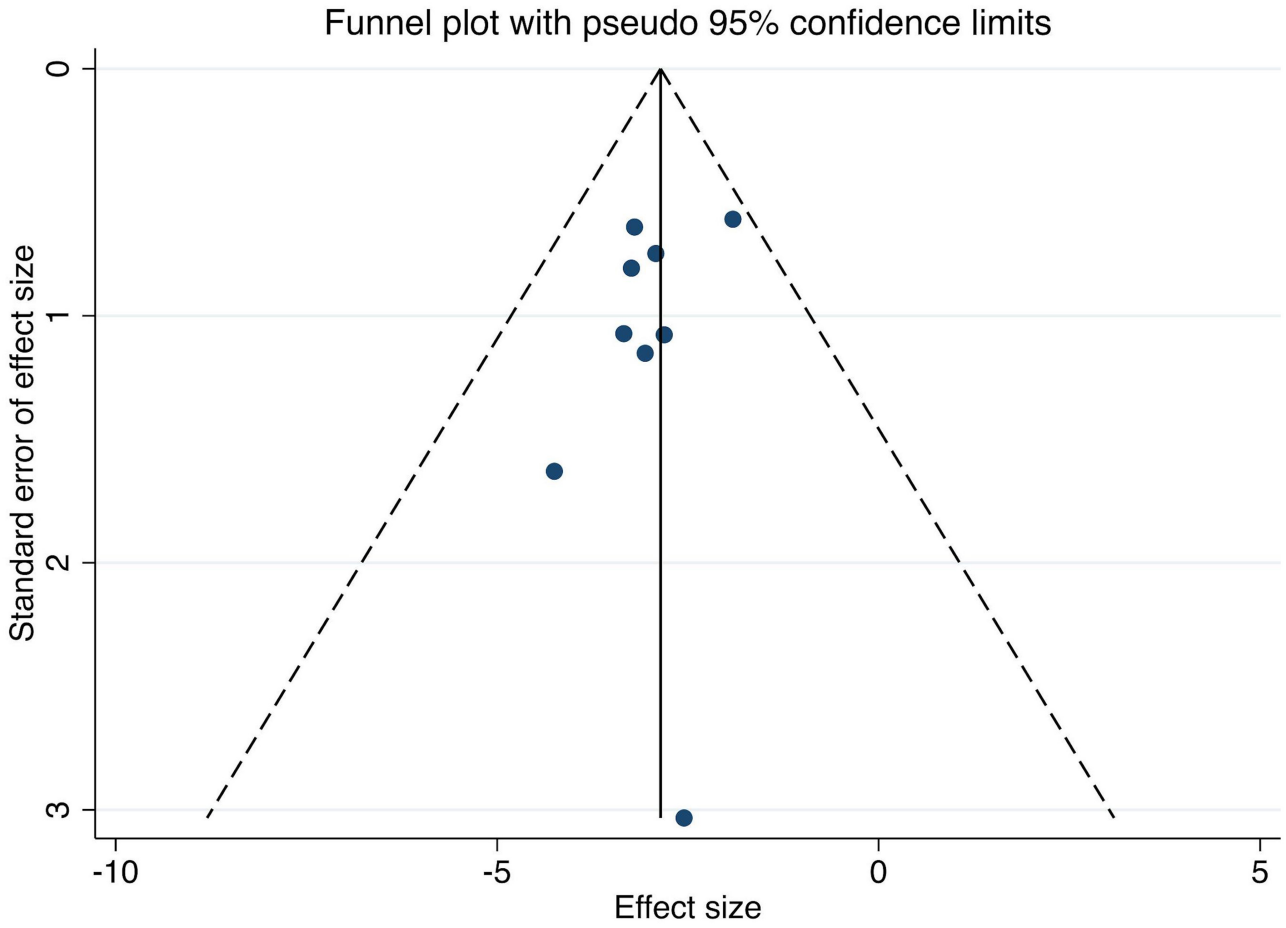
Funnel plot assessing publication bias for studies reporting MASI score. The plot shows the SMD of each study against its standard error. Symmetrical distribution around the pooled effect size (vertical line) suggests a low risk of publication bias. MASI, Melasma Area and Severity Index.

**Figure 10:**
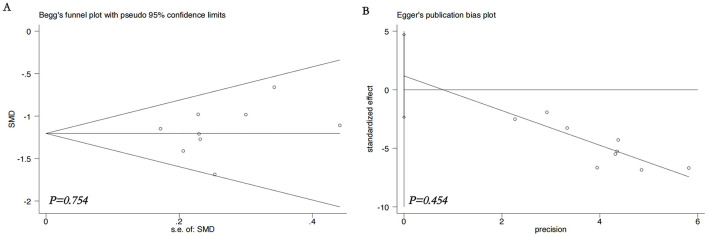
Statistical tests for publication bias on MASI score. **(A)** Begg's funnel plot, **(B)** Egger's funnel plot. Non-significant *p* values (>0.05) from both tests indicate no strong statistical evidence of publication bias. MASI, Melasma Area and Severity Index; SMD, standardized mean difference.

**Figure 11:**
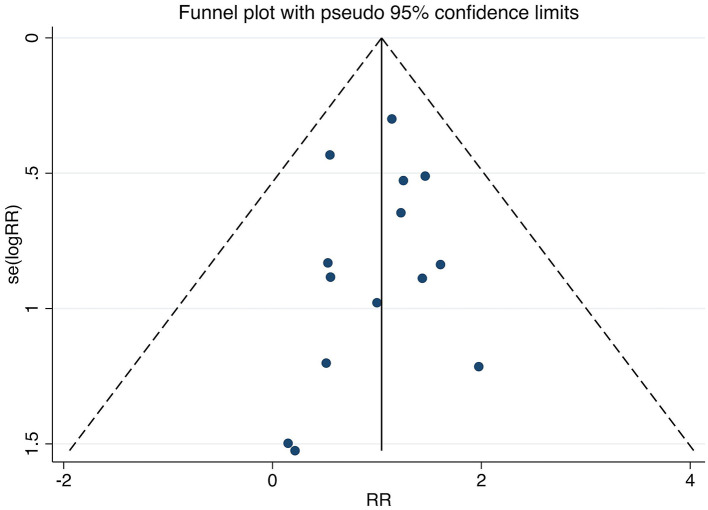
Funnel plot assessing publication bias for studies reporting adverse reactions rate. The plot shows the RR of each study against its standard error. RR, relative risk.

**Figure 12:**
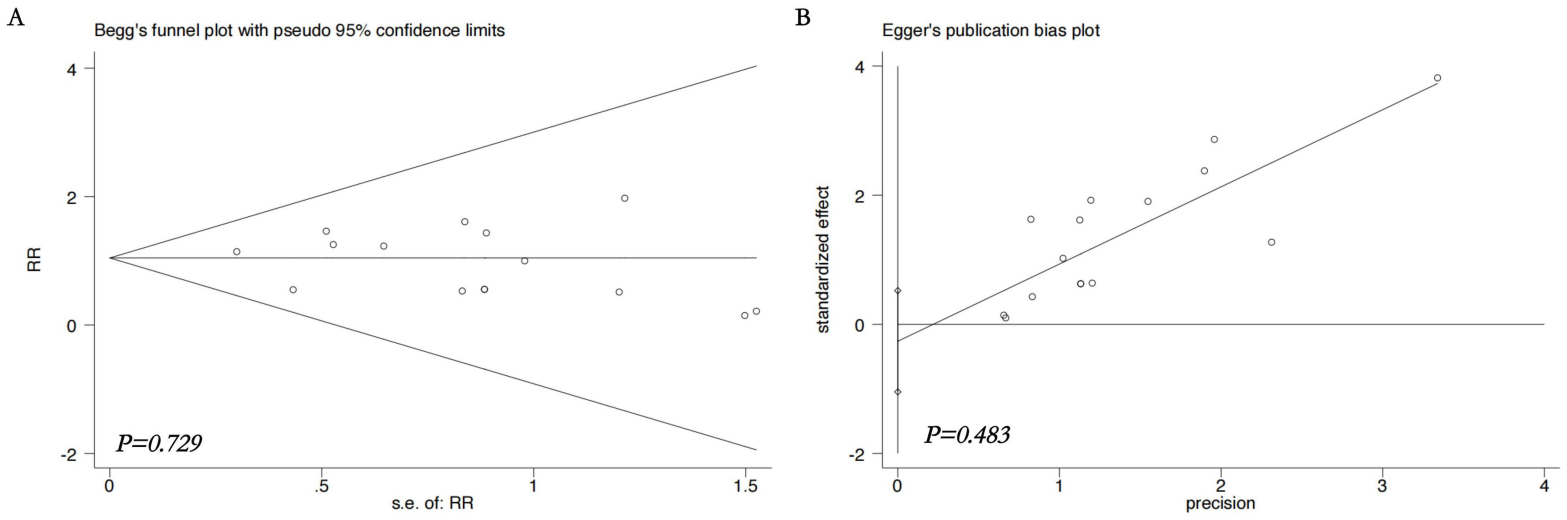
Statistical tests for publication bias on adverse reactions rate. **(A)** Begg's funnel plot, **(B)** Egger's funnel plot. Non-significant *p* values (>0.05) from both tests indicate no strong statistical evidence of publication bias. RR, relative risk.

Publication bias was evaluated for the primary outcomes (MASI score and adverse reaction rates). Visual inspection of the funnel plots suggested approximate symmetry, which was supported by non-significant results from both Begg's test (all *p* > 0.05) and Egger's test (all *p* > 0.05). The funnel plots are presented in Figures 9–12.

## 5 GRADE evidence quality assessment

The GRADE evaluation showed moderate-quality evidence (⊕⊕⊕○) for most outcomes—MASI improvement, effectiveness, adverse reactions, and satisfaction—but low-quality evidence (⊕⊕○○) for recurrence rates. The limited number of studies reporting recurrence rates is a key reason for the downgrading of the evidence quality for this outcome. The complete evidence profile is provided in Table 3.

**Table 3:** GRADE evidence profile for outcomes of combined QS laser and IPL therapy for melasma.

**Outcome measure**	**Number of included studies**	**Primary study design**	**Evidence quality assessment**	**Overall quality of evidence**
MASI score	9	RCTs	No serious risk of bias, inconsistency, or indirectness; downgraded for imprecision (−1)	Moderate ⊕⊕⊕○
Clinical efficacy rate	26	Predominantly RCTs	No serious risk of bias, inconsistency, or indirectness; downgraded for imprecision (−1)	Moderate ⊕⊕⊕○
Adverse reactions rate	15	Predominantly RCTs,	No serious risk of bias or indirectness; downgraded for inconsistency (−1) and imprecision (−1)	Moderate ⊕⊕⊕○
Recurrence rate	4	Predominantly RCTs	No serious risk of bias, inconsistency, or indirectness; downgraded for imprecision (−1); Publication bias undetected	Low ⊕⊕○○
Patient satisfaction rate	6	Predominantly RCTs	No serious risk of bias, inconsistency, or indirectness; downgraded for imprecision (−1); publication bias undetected	Moderate ⊕⊕⊕○

Evidence Quality Symbols: High (⊕⊕⊕⊕), Moderate (⊕⊕⊕○), Low (⊕⊕○○), Very Low (⊕○○○). Downgrading reasons included imprecision and inconsistency. GRADE, grading of recommendations assessment, development and evaluation; IPL, intense pulsed light; MASI, melasma area and severity index; QS, Q-Switched laser; RCT, randomized controlled trial.

## 6 Discussion

Melasma is a disfiguring facial dermatosis that causes substantial psychological distress and economic burdens for affected individuals. This highlights the persistent need for more effective and durable treatment options. While numerous therapeutic modalities exist, each demonstrates distinct limitations that complicate long-term management. In recent years, laser and light-based interventions—particularly Q-switched (QS) lasers and intense pulsed light (IPL)—have gained increasing clinical interest.

This systematic review and meta-analysis provides the first quantitative evaluation of the efficacy and safety of combined QS laser and IPL therapy compared to monotherapy for melasma. The results indicate that compared to the monotherapy groups, participants receiving combination therapy achieved significantly greater improvement in the Melasma Area and Severity Index (MASI) scores and a higher clinical response rate, along with a reduction in recurrence. Furthermore, no significant differences were observed between the two groups in terms of adverse event rates or patient satisfaction. This discussion will analyze the clinical implications, explore the potential mechanisms underlying the enhanced efficacy of this combined approach, and consider its clinical significance in the management of this complex dermatological condition.

Building on the observed effectiveness of combination therapy in lowering MASI scores, further investigation into the underlying mechanisms was conducted. The improved outcomes may result from the combined effects of QS lasers and IPL, which work through heat and sound-based mechanisms. In the included studies, QS lasers were mainly Q-switched Nd:YAG lasers (1,064 nm), with the fluence ranging from 0.8 to 4.0 J/cm^2^, pulse duration of 5–20 ns, spot size of 6–10 mm, 3–6 treatment sessions and 2–4 weeks interval between sessions. IPL was usually set at the wavelength of 560–1,200 nm with cut-off filters, fluence of 15–30 J/cm^2^, pulse duration of 3–10 ms (mostly double or triple pulses), spot size of 8–15 mm, 4–8 treatment sessions and 2–4 weeks interval. Most combination therapy protocols applied alternating regimens, and all included studies adopted contact cooling during treatment and recommended daily use of broad-spectrum sunscreen (SPF ≥30, PA + + + ) post-treatment to minimize photodamage. Notably, the included studies showed low heterogeneity, supporting the reliability of the pooled results; however, a parameter-level meta-analysis was not feasible due to incomplete reporting of treatment parameters in individual studies (4, 8, 42, 43). The Q-switched Nd:YAG laser, especially when applied at low fluence, effectively disrupts melanosomes and selectively targets melanin-rich cells through a process known as photothermolysis (42). Meanwhile, IPL works by targeting both epidermal and superficial dermal pigmentation, utilizing selective absorption by melanin and hemoglobin chromophores (43, 44). Beyond pigment-targeting, IPL can modulate inflammatory mediators, reduce mast cell infiltration, downregulate pro-angiogenic pathways, remodel dermal extracellular matrix, and restore basement membrane integrity—key effects to counteract the chronic persistence and recurrence of melasma, as well as improve vascular abnormalities and dermal photoaging-related changes (8, 45–47). In contrast, QS laser monotherapy only focuses on melanin clearance, lacking regulatory effects on dermal stroma, vascular structures and inflammatory responses associated with melasma, which accounts for its limited efficacy and relatively high recurrence rate (4, 10, 48). This complementary mechanism of the two modalities not only achieves synergistic therapeutic efficacy but also provides a mechanistic basis for its anti-recurrence potential (8, 46, 47), which is verified by reflectance confocal microscopy and neural network evaluations showing significant and sustained MASI score reductions (8, 46). Unlike monotherapies (QS laser or IPL) with moderate efficacy (usually below 60%), combined therapy targets multiple pathological pathways (pigment metabolism, vascular dysregulation and inflammatory cascades) simultaneously to achieve higher treatment response rates, which is consistent with existing academic consensus (4, 6, 8, 10, 49). Molecular and imaging data further confirm that this combined strategy reduces pigmentation while optimizing the local skin microenvironment, with its superior efficacy rooted in enhanced pigment clearance and blocked chronic inflammatory and vascular cycles involved in melasma pathogenesis (45, 47, 48).

Notably, variations in therapeutic efficacy among the included studies may be attributed to differences in device settings, patient characteristics, and lesion duration. It should be emphasized that melasma is a chronic and recurrent cutaneous disorder, and at least 12 months of long-term follow-up is indispensable to confirm the durability of treatment effects. However, current evidence has clear limitations: the number of studies specifically evaluating recurrence rates is limited, and all studies only provided short-term follow-up data. Based on these short-term data, it is important to avoid overstating the long-term anti-recurrence benefits of combination therapy. Whether combination therapy possesses a definitive long-term anti-recurrence advantage still requires verification through future high-quality prospective studies with extended follow-up (2, 4, 47). Collectively, existing findings support that simultaneous intervention of both pigment-related and non-pigment-related pathogenic pathways is key to achieving remission (4, 6, 47), although the certainty of its long-term effects needs further confirmation.

This study found that combination and monotherapy regimens have comparable safety profiles regarding adverse reaction rates. This finding is significant, especially given concerns about cumulative photothermal damage and post-inflammatory hyperpigmentation (PIH). However, our results regarding safety contrast with the findings of Guan et al. (13), who observe a higher rate of PIH. This discrepancy may result from differences in treatment parameters, patient characteristics—including Fitzpatrick skin type—and operator experience. Previous studies show that using QS lasers and IPL at appropriate fluences and intervals causes minimal adverse effects—mainly transient erythema or mild irritation—and rarely leads to serious complications (49, 50). Notably, the risk of adverse events seems to be more closely associated with the operator's technique, energy settings, and the patient's skin phototype rather than the use of combination therapies themselves (8). Because combined treatment protocols do not increase complication rates, this supports the idea that carefully adjusting treatment parameters can maintain safety while improving efficacy. Moreover, IPL's anti-inflammatory properties may help reduce irritation caused by laser treatments (51). Overall, these findings confirm that combination therapy can be implemented without a corresponding rise in safety risks.

Although the combined therapy group showed superior objective outcomes, such as a significant reduction in MASI scores and higher clinical efficacy, patient satisfaction did not differ significantly between the combined and monotherapy groups. This clinically meaningful discrepancy between objective efficacy and subjective satisfaction is closely related to the clinical value and patient compliance of combined therapy, warranting further in-depth discussion. Firstly, psychosocial factors play a core role: melasma patients often experience long-term psychological distress, including anxiety and social avoidance, which can reduce their recognition of objective treatment improvements despite significant MASI score reductions (1, 2). Secondly, baseline treatment expectations are critical: patients with unrealistic high expectations for complete depigmentation have low satisfaction with partial efficacy, while those who establish reasonable expectations through standardized pre-treatment counseling have higher subjective recognition of the same curative effect (2, 5). Thirdly, limitations of assessment tools further aggravate this discrepancy. Most included studies used simple single-item scales (e.g., satisfied, general, dissatisfied) without multidimensional evaluation of transient skin discomfort (redness, dryness, itching) during treatment and recovery, thus failing to fully capture the comprehensive treatment experience. Additionally, the beneficial improvements of combined therapy on dermal vascular abnormalities and inflammatory microenvironment are invisible to patients, which cannot be reflected in subjective satisfaction scores and lead to a disconnection between objective and subjective perceptions (4, 7). In summary, patient satisfaction depends not only on clinical indicators like MASI reduction and efficacy rate, but also on factors such as psychosocial status, disease severity at baseline, and treatment expectations (1, 2, 5). Even with significant pigment clearance, patients' worries about recurrence, recovery duration and mild transient adverse reactions will negatively affect overall satisfaction (4, 49), which highlights the necessity of thorough pre-treatment counseling, effective expectation management, and the importance of developing validated multidimensional patient-reported outcome metrics in future clinical trials (2, 5).

### 6.1 Limitations

This study has several critical limitations that need to be comprehensively addressed. Firstly, although the included studies exhibited low heterogeneity, ensuring the reliability of pooled results, they were geographically concentrated, with most originating from China and East Asia. The dominant Fitzpatrick skin types in this region (III–IV) differ significantly from Caucasian (I–II) and African (V–VI) populations in melanin content, epidermal thickness, and susceptibility to photodamage-induced adverse reactions (2, 4). Moreover, regional differences in treatment parameters, clinical guidelines, operator proficiency, and patient compliance further limit the global generalizability and external validity of these findings. Secondly, all included studies have short follow-up durations (3–12 months for most, maximum 12 months); for the chronic recurrent melasma, at least 12 months of long-term follow-up is the core prerequisite for evaluating treatment durability (2, 48), and the lack of such long-term data fails to confirm the sustained efficacy and definite anti-recurrence value of combined therapy, which helps avoid overstating its long-term clinical benefits. Thirdly, the aggregate sample size, though sufficient for the primary analysis, was not large enough to fully capture the demographic and clinical heterogeneity of melasma. Additionally, the lack of complete Fitzpatrick skin type data across all included studies precluded planned subgroup analyses based on skin phototype. Fourthly, The scarcity of mechanistic studies on the synergistic effect of combined therapy limits our in-depth understanding of the underlying core biological processes. This limitation hinders further optimization and precise clinical application of the regimen. In addition, incomplete reporting of treatment parameters in included studies made parameter-level meta-analysis unfeasible, and the single-item scales for patient satisfaction assessment failed to comprehensively reflect the overall treatment experience, leading to the inability to fully evaluate the subjective benefits of combined therapy.

## 7 Conclusion

This study confirms that combined QS laser and IPL therapy is more effective than monotherapies for melasma. It provides better clinical outcomes, a lower short-term recurrence rate, and an acceptable safety profile. Nevertheless, since the findings are based on short follow-up data from a limited geographic region, their long-term applicability and generalizability are limited. This combined regimen is a promising method to improve melasma treatment; however, its long-term therapeutic value requires further verification.

### 7.1 Future perspectives

To build upon these findings, future research should prioritize long-term follow-up studies exceeding 12 months to substantiate the anti-recurrence benefit. Furthermore, investigations into the underlying mechanisms and studies incorporating Fitzpatrick skin type into the evaluation framework are needed. It is also recommended that studies standardize laser and IPL parameter reporting and use multidimensional patient-reported outcome measures. Finally, conducting international multi-center studies involving diverse populations will be crucial to improve the external validity of future findings and maximize patient benefit.

## Data Availability

The original contributions presented in the study are included in the article/supplementary material, further inquiries can be directed to the corresponding authors.
